# The Relationship Among Mentalization, Mindfulness, Working Memory, and Schizotypal Personality Traits in the General Population

**DOI:** 10.3389/fpsyg.2022.682889

**Published:** 2022-05-02

**Authors:** Edina Török, Szabolcs Kéri

**Affiliations:** ^1^Department of Cognitive Science, Budapest University of Technology and Economics, Budapest, Hungary; ^2^Nyírő Gyula National Institute of Psychiatry and Addictions, Budapest, Hungary; ^3^Department of Physiology, University of Szeged, Szeged, Hungary

**Keywords:** mindfulness, mentalization, working memory, schizotypy, theory of mind

## Abstract

Individuals with high schizotypal traits are less able to observe, describe, and monitor inner feelings, thoughts, and experiences, commonly referred to as mindfulness and mentalization. High schizotypy is also associated with impaired working memory (WM). However, the relationship among mindfulness, mentalization, WM, and schizotypal traits is unknown. Three hundred individuals from the community (mean age: 38.0 years, *SD* = 10.5; 49.3% women) completed questionnaires examining schizotypal traits, mindfulness, and mentalization and performed working memory tasks. Results revealed that mentalization was a general predictor of schizotypal traits, including unusual experiences, cognitive disorganization, introverted anhedonia, and impulsive nonconformity, when the effect of mindfulness and working memory was controlled. We also found a positive correlation between mindfulness and mentalization. Low mindfulness and mentalization performances were associated with high schizotypy. However, poor working memory was only weakly linked to cognitive disorganization and introverted anhedonia. These findings suggest that weak mentalization is a core feature of schizotypy independent of mindfulness and working memory.

## Introduction

Currently, the dimensional approach to mental illness receives special attention. A typical example is the odd and eccentric nature of schizotypal personality, which exhibits some similarities with schizophrenia-spectrum disorders (Raine et al., 1994; Mason et al., 2005; Nelson et al., 2013; Ettinger et al., 2014; Cohen et al., 2015). People with high levels of schizotypal traits are characterized by unusual experiences (illusions and perceptual distortions), bizarre beliefs, loosened associations, less conventional social behavior, and isolation. First, unusual experiences, also known as the cognitive-perceptual dimension of schizotypy, refer to anomalous perceptual experiences, overvalued and unusual thoughts, and suspiciousness. In schizophrenia patients, these traits turn into the symptoms of frank hallucinations and delusions. Second, introverted anhedonia, also termed the interpersonal dimension of schizotypy, involves decreased interest in pleasurable social activity, diminished feelings, and reduced volition. These schizotypal traits are reminiscent of the negative symptoms of schizophrenia, including blunted affect, social withdrawal, avolition, and alogia. Third, individuals with disorganized schizotypy display derailed thinking, loosened associations, and eccentric behavior, which are milder variants of incoherent thinking and grossly disorganized behavior in schizophrenia. Finally, eccentric and odd behavior often pairs with impulsive nonconformity in schizotypy: weak attunement to social conventions, poor impulse control, and deficient emotion regulation (Raine et al., 1994; Mason et al., 2005; Rosell et al., 2014).

Adaptive personality functioning is founded on the human ability to build an integrated and nuanced internal model of the social world, experience and regulate thoughts, feelings, and impulses, and understand self-other interactions in terms of mental states and intentions. As seen from the above-described features of schizotypy, one can assume a marked alteration of social cognitive functions in individuals with high schizotypy. Dating back to fundamental psychoanalytic theories of personality and mind, two leading concepts in contemporary psychology aim to characterize the experience and regulation of mental states and their differentiation from the external world: mentalization and mindfulness (Klein, 1946; Bion, 1962; Fonagy et al., 2002; Choi-Kain and Gunderson, 2008; Kernberg, 2011). Although these concepts share several features, there also are multiple distinctive features.

Mentalization refers to the interpretation of other- and self-related behavior in terms of mental states (intentions, beliefs, and desires; Dennett, 1987; Fletcher et al., 1995; Fonagy et al., 1997, 2002, 2016; Bateman and Fonagy, 2010). Most scholars accept the view that mentalization is not a unitary construct: it can be automatic (preconscious) or controlled (conscious), self-focused and other-centered, driven by internal or external features of self and others, and cognitive or affective (Fonagy and Luyten, 2009). In addition to understanding the social world and setting the boundary between external reality and the inner world (i.e., psychic equivalence and pretend modes), mentalization is critical in reflective self-representation, emotional awareness, and affective regulation. From a developmental perspective, there is a strong link between attachment to important others and mentalization, a foundation of stable self-identity (Main, 1991; Fonagy et al., 2002; Karterud and Kongerslev, 2019). This relationship between mentalization and developmental attachment can explain why mentalization is socially and interpersonally context-dependent and denote the relationship between two persons.

Mindfulness, which derives from Buddhist meditative tradition and is widely accepted and popularized in Western psychology, also embraces self-observation (Kabat-Zinn, 1996; Falkenstrom, 2003; Bishop et al., 2004; Chi et al., 2018). However, in contrast to reflective mentalization on past experiences, individuals practicing mindfulness focus their attention on the present moment without any cognitive effort and preoccupation to readily observe and accept percepts, thoughts, and feelings as they appear and vanish. Mindfulness is conscious and explicit, related to the self (inner experiences of thoughts, feelings, images, and bodily sensations) and sometimes external events (sounds and sights). Thus, the main difference between mentalization and mindfulness is that the latter does not deal with other individuals’ inner states and emotions (Choi-Kain and Gunderson, 2008; Falkenström et al., 2014; Marszal and Górska, 2015). Moreover, explicit mentalization requires intensive cognitive elaboration to maintain, understand, and verbalize inner mental states, referring to past experiences and future perspectives, going beyond the mere moment-by-moment observation and attention of mindfulness. However, empirical evidence shows that scores on scales measuring mentalization and mindfulness share considerable variance (Falkenström et al., 2014; Marszal and Górska, 2015). Mindfulness is a foundation so as to consciously contain internal representations as a starting point of mature mentalization. Marszal and Górska (2015) emphasized that Kernberg (2011) had compared mindfulness with containment, which is essential for the regulation of aggressive and threatening feelings related to the self and others. In other words, the primary purpose of mindfulness is the acceptance of emotions without acting them out in the external world. Mature mentalization serving emotion regulation is possible only if there is an appropriate containing mindfulness function for intensive and ambivalent emotions (Kernberg, 2011). Overall, mindfulness facilitates the acceptance of experiences without prejudice and action, and indirectly affects self-regulation as a gateway to genuine mentalization (Fossati et al., 2012).

Altered mentalization and mindfulness may contribute to schizotypy and schizophrenia-spectrum disorders *via* dysfunctional attentive appreciation, experience, and metacognitive reflection on mental states (Lysaker et al., 2021). In patients with schizophrenia, impaired mentalization substantially contributes to social dysfunctions, and its neurocognitive bases may provide new insight into the development of innovative psychotherapeutic and pharmacological methods (Dimopoulou et al., 2017). Moreover, mentalization is markedly impaired even in patients with first-episode psychosis; in unaffected relatives of schizophrenia patients and individuals with ultra-high risk for psychosis, mentalization performance is intermediate between first-episode psychosis and healthy individuals (Bora and Pantelis, 2013).

Several studies attempted to characterize the association between mentalization and schizotypy, but the results remained controversial (Langdon and Coltheart, 1999; Henry et al., 2008; Morrison et al., 2013; Acosta et al., 2019). Initial evidence suggested a selective mentalization deficit in high schizotypal individuals from the general population that might contribute to psychotic-like traits, forming a continuum with clinically diagnosed schizophrenia (Langdon and Coltheart, 1999). Others emphasized that the mentalization deficit was not related to a specific schizotypal trait (Morrison et al., 2013). Furthermore, some findings indicated only a weak association between unusual experiences and mentalization without a global impairment of mentalization in people with high schizotypy (Pickup, 2006). Therefore, the intuitive assumption that general schizotypy is associated with less efficient mentalization has been challenged (Jahshan and Sergi, 2007; Fernyhough et al., 2008; Barragan et al., 2011; Kallai et al., 2019; Wastler and Lenzenweger, 2019). Attempts to separately investigate the specific link between negative and positive schizotypy and mentalization yielded mixed results without a generally accepted conclusion (Pickup, 2006; Fernyhough et al., 2008; Henry et al., 2008; Barragan et al., 2011; Gooding and Pflum, 2011). Moreover, some studies indicated over-mentalizing in positive schizotypy, leading to an exaggerated attribution of mental states to others, which may form the basis of overvalued ideas, referential thinking, and delusional inferences (Fyfe et al., 2008; Wastler and Lenzenweger, 2019). Mentalization would be strongly linked to cognitive disorganization because lack of concentration and social anxiety constitute cognitive disorganization. Furthermore, social rejection affects cognitive disorganization more than unusual experiences and introvertive anhedonia (Premkumar and Kumari, 2022). Thus, mentalization declines when there is a high level of social anxiety (Ballespí et al., 2021).

In contrast to mentalization, the literature provides little information on the relationship between schizotypy and mindfulness. Bronchain and Chabrol (2020) used a network theory approach to explore the relationship between dispositional mindfulness and schizotypy. The findings indicated a strong relationship between weakened mindfulness (decreased capacity to describe inner experiences, acting with awareness, and nonjudgment) and interpersonal schizotypy. According to the authors, alterations in mindfulness may lead to a diminished capacity to experience emotions and dysfunctional self-agency in schizotypy (Bronchain and Chabrol, 2020). From another point of view, mindfulness meditation does not enhance schizotypal traits in general: individuals who regularly practice mindfulness meditation exhibited reduced suspiciousness, low social anxiety, but they also displayed enhanced magical thinking (Antonova et al., 2016). Higher dispositional mindfulness may turn positive schizotypy into creativity and leads to lower suspicion (Mcdonald et al., 2021). Surprisingly, one study found intact dispositional mindfulness in schizophrenia (López-Del-Hoyo et al., 2019).

To our knowledge, no attempts were made to investigate the relationship among mentalization, mindfulness, and dimensions of schizotypy (unusual experiences, cognitive disorganization, introverted anhedonia, and impulsive nonconformity) in the same population. Therefore, the latent interaction between mentalization and mindfulness concerning schizotypy is unknown. An additional potential confounding factor in such analyses is working memory (WM), which refers to a short-term and limited capacity system that serves the retaining of behaviorally relevant information. There is some empirical evidence for the association between worse WM and higher schizotypy (Ettinger et al., 2015; Siddi et al., 2017). Working memory deficits are associated with positive and negative schizotypy and may contribute to dysfunctional psychosocial adaptation (Ettinger et al., 2015). As a general impairment in top-down attentional control, working memory deficits may be related to the weakened maintenance, control, and manipulation of internal representations implicated in mindfulness and mentalization. Although the traditional view suggests an inverse relationship between task-oriented problem solving (working memory) and introspective reflection on intentions and feelings (mentalization), demanding social cognition simultaneously involves both mentalization and working memory at the behavioral and neuronal level (Meyer and Lieberman, 2012). In patients with schizophrenia, abnormal activation of lateral prefrontal areas, which are canonical regions engaged for working memory, correlated with impaired Theory of Mind (ToM) performance (Pu et al., 2016). Therefore, working memory may be critical in the relationship between mentalization and schizotypal traits: dysfunctional mentalization may result from a lessened capacity to actively retain information in individuals with high schizotypy (Kocsis-Bogar et al., 2017).

Therefore, we presumed the following hypothesis: (i) high schizotypal traits are associated with poor mentalization and mindfulness; (ii) there is a significant correlation between mentalization and mindfulness; and (iii) working memory impairment can explain a significant amount of variance in schizotypy, mentalization, and mindfulness.

## Materials and Methods

### Procedure

We recruited the participants from the general population *via* social media advertisement and random digit dialing. In those who decided to visit the laboratory (participation rate of all people invited as: 63%), we first registered the essential demographic characteristics and administered a clinical interview for mental disorders. Second, the participants completed three questionnaires (schizotypy, mentalization, and dispositional mindfulness) and a set of working memory tests (digit span, letter-number sequencing, and arithmetic). The authors and qualified research assistants personally administered the scales and working memory tests in the laboratory. We used the standard and validated Hungarian version of the scales and tests (Balázs et al., 1998; Rózsa et al., 2006; Simor et al., 2013; Kocsis-Bogár et al., 2016; Fekete et al., 2019).

### Participants

We assessed 300 non-clinical individuals (152 men, 148 women; all Caucasian; 72% urban population; and 18% suffering from chronic illness), aged 18–70 years. The average age was 38.0 years (*SD* = 10.5). The average duration of education was 11.2 years (*SD* = 4.6). We used digital social media advertisement and random digit dialing—recruited postal survey to obtain a representative sample for age, gender, education, income, rural and urban geography, and perceived health (all Cramer values of *V* < 0.1; Shaver et al., 2019). Following a detailed description of the study, we obtained written informed consent from the participants. The study was reviewed and approved by the National Medical Research Council (ETT-TUKEB 18814, Budapest, Hungary). We performed all procedures according to the relevant guidelines, regulations, and the Declaration of Helsinki.

### Mini International Neuropsychiatric Interview 7.0

The mini international neuropsychiatric interview 7.0 (MINI 7.0) is a brief and structured clinical interview for the 17 most common disorders defined in the Diagnostic and Statistical Manual of Mental Disorders-5 (DSM-5; American Psychiatric Association, 2013; Sheehan, 2015). The administration time is 15–20 min. The MINI 7.0 is validated against the DSM-5-CV (Structured Clinical Interview for DSM-5 Disorders—Clinician Version) in Hungarian (First et al., 2016).

### Oxford-Liverpool Inventory of Feelings and Experiences, Short Version

The Oxford-liverpool inventory of feelings and experiences, short version (sO-LIFE) consists of 43 dichotomous items (yes—no responses) measuring the four dimensions of schizotypal traits: Unusual Experiences (unusual perceptual experiences and non-ordinary beliefs; 12 items), Cognitive Disorganization (loosening of associations, lessened ability to focus attention, and social anxiety; 11 items), Impulsive Nonconformity (impulsivity and weak adherence to social norms; 10 items), and Introvertive Anhedonia (reduced ability to experience pleasure and decreased motivation to participate in social activities; 10 items; Mason et al., 2005; Mason and Claridge, 2006; Kocsis-Bogár et al., 2016). Participants with high scores exhibit pronounced schizotypy. The internal consistency of Unusual Experiences and Cognitive Disorganization dimensions were good (*α* > 0.8). In the case of Impulsive Nonconformity and Introvertive Anhedonia, we observed acceptable alpha-values (0.85 > *α* > 0.65).

### Mentalization Questionnaire

The mentalization questionnaire (MZQ) is a 15-item self-report questionnaire. The items focus on self-reflection (e.g., “Most of the time it is better not to feel anything.”), emotional awareness (e.g., “Often I do not even know what is happening inside of me.”), psychic equivalence mode (e.g., “If I expect to be criticized or offended, my fear increases more and more.”), and affect regulation (e.g., “Often I cannot control my feelings.”). Each item is rated on a 0- to 4-point scale (0 – totally disagree; 4 – totally agree; range of full-scale score: 0–60). Individuals with weak mentalization achieve high scores (Hausberg et al., 2012; Fekete et al., 2019).

### Mindful Attention Awareness Scale

The mindful attention awareness scale (MAAS) is a self-report questionnaire consisting of 15 items to measure dispositional mindfulness, which refers to openness, receptiveness, and attention to the present moment (sample items: “I could be experiencing some emotion and not be conscious of it until sometime later.”; “I break or spill things because of carelessness, not paying attention, or thinking of something else.”; and “It seems I am ‘running on automatic’, without much awareness of what I am doing.”; Brown and Ryan, 2003; Simor et al., 2013). Participants rate how frequently they experience an item (1 – almost always; 6 – never). High scores reflect effective mindfulness (range: 15–90). The MAAS has good psychometric properties (internal consistency: *α* > 0.85; intra-class correlation for test–retest scores: 0.84).

### Working Memory Index

We used the Wechsler Adult Intelligence Scale-III (WAIS-III) WM index consisting of three tests (Wechsler, 1997; Lange, 2011). The Digit Span test, assessing verbal short-term memory and attention, consists of two parts. In the digits forward condition, participants repeat a previously exposed series of numbers. In the digits reversed condition, participants say the digits back in reverse order. In the Letter-Number Sequencing test, participants repeat letters and numbers with the letters in alphabetical order and the numbers in numerical order. Finally, the Arithmetic test comprises mental arithmetic questions (e.g., “If Jo has 12 buns, he then eats three and gives four away how many does he have left?”).

### Data Analysis

We used STATISTICA 13.5 (TIBCO, Palo Alto) and G*power 3.1.9.2 (UCLA, Institute for Digital Research and Education, Statistical Consulting). In the descriptive statistics, we calculated means, SDs, range, kurtosis, and skewness. Raw data were examined for normal distribution with Kolmogorov–Smirnov tests. First, bivariate correlations were conducted (Pearson’s product–moment correlation coefficients) between sO-LIFE, MZQ, MAAS, and WAIS-III-WM scores. The significance level was Bonferroni-corrected [correlation analysis: *p* < 0.001 (0.05/49); regression analysis: *p* < 0.003 (0.05/15)]. Second, multiple regression analyses were performed, controlled for age and gender, for the four dimensions of sO-LIFE schizotypy (Unusual Experiences, Cognitive Disorganization, Introverted Anhedonia, and Impulsive Nonconformity). First, we proposed that mindfulness is the starting point of mentalization; therefore, it was the first predictor. Next, we added working memory to the model to control the non-specific effect of active maintenance of information. Finally, mentalization was incorporated into the regression model to test the covariance and predictive power controlled for mindfulness and working memory. Therefore, in the first step, MAAS (mindfulness) was entered as a predictor of the schizotypy dimension, followed by WAIS-III-WM (working memory) and MZQ (mentalization). To obtain a statistical power of 80%, we enrolled 300 individuals. The level of statistical significance was *α* < 0.05.

## Results

### Descriptive Statistics and Correlation Analysis

Means, SDs, and ranges for schizotypal traits, mindfulness, mentalization, and working memory scores are depicted in Table 1. High Unusual Experiences, Cognitive Disorganization, and Introverted Anhedonia were associated with low mentalization and mindfulness abilities (*p*s < 0.001, Table 2). Individuals with less effective mindfulness also exhibited a lower capacity to mentalize (*r* = −0.58, *p* < 0.001, Table 2). Cognitive Disorganization, Introverted Anhedonia, and mentalization weakly and negatively correlated with working memory (*p* < 0.05), whereas we found a more pronounced association between mindfulness and working memory (*p* < 0.001, Table 2).

**Table 1:** Descriptive data for schizotypal personality traits, mindfulness, mentalization, and working memory (WM).

	Mean	Standard deviation	Range
sO-LIFE	
Unusual experiences	2.9	2.0	0–9
Cognitive disorganization	2.8	1.9	0–9
Introverted anhedonia	3.6	2.6	0–12
Impulsive nonconformity	2.2	1.7	0–8
MAAS	52.6	14.7	15–90
MZQ	25.3	13.6	0–55
WAIS-III WM	103.1	10.4	85–134

*sO-LIFE, oxford-liverpool inventory of feelings and experiences, short version; MAAS, mindful attention awareness scale; MZQ, mentalization questionnaire; and WAIS-III WM, Wechsler adult intelligence scale-III (WAIS-III) working memory index*.

**Table 2:** Correlations between schizotypal traits, mindfulness, mentalization, and WM (*N* = 300).

	1	2	3	4	5	6	7
1. Unusual experiences		0.50^***^	0.41^***^	0.25^***^	0.38^***^	−0.22^***^	−0.11
2. Cognitive disorganization	0.50^***^		0.34^***^	0.23^***^	0.40^***^	−0.32^***^	−0.15^*^
3. Introverted anhedonia	0.41^***^	0.34^***^		−0.14^*^	0.32^***^	−0.25^***^	−0.13^*^
4. Impulsive nonconformity	0.25^***^	0.23^***^	−0.14^*^		0.22^***^	−0.05	0.04
5. MZQ	0.38^***^	0.40^***^	0.32^***^	0.22^***^		−0.58^***^	−0.18^**^
6. MAAS	−0.22^***^	−0.32^***^	−0.25^***^	−0.05	−0.58^***^		0.40^***^
7. WAIS-III WM	−0.11	−0.15^*^	−0.13^*^	0.04	−0.18^**^	0.40^***^	

*sO-LIFE, oxford-liverpool inventory of feelings and experiences, short version; MAAS, mindful attention awareness scale; MZQ, mentalization questionnaire; and WAIS-III WM, Wechsler adult intelligence scale-III (WAIS-III) working memory index*.

* *p* *< 0.05;*

** *p* *< 0.01;*

*** a < 0.001 (*** = Bonferroni-corrected threshold).

### Predictors of Total sO-LIFE Scores

In the first step of the analysis, mindfulness significantly predicted the total sO-LIFE score (*R*^2^ = 0.10, *β* = −0.32, *SE* = 0.05, *p* < 0.001). We then added the working memory index to the model, which resulted in no significant changes (*R*^2^ = 0.10; working memory: *β* = −0.02, *SE* = 0.06, *p* = 0.80; mindfulness: *β* = −0.32, *SE* = 0.06, *p* < 0.001). In the final step of the analysis, we included mentalization in the model, which appeared as the sole significant predictor of the total sO-LIFE score (*R*^2^ = 0.25; working memory: *β* = −0.05, *SE* = 0.05, *p* = 0.40; mindfulness: *β* = −0.03, *SE* = 0.07, *p* = 0.61; and mentalization: *β* = 0.47, *SE* = 0.06, *p* < 0.001).

### Predictors of Unusual Experiences

In the first regression model, we found that mindfulness significantly predicted the Unusual Experiences (*R*^2^ = 0.06, *β* = −0.22, *SE* = 0.06, *p* < 0.01, not significant after correction for multiple comparisons). When the working memory index was added to the model, we did not observe significant changes (*R*^2^ = 0.06; working memory: *β* = −0.03, *SE* = 0.06, *p* = 0.58; mindfulness: *β* = −0.21, *SE* = 0.06, *p* < 0.01). In the final model, mentalization was included, and it emerged as the sole significant predictor of Unusual Experiences (*R*^2^ = 0.15; working memory: *β* = −0.06, *SE* = −0.06, *p* = 0.33; mindfulness: *β* = −0.01, *SE* = 0.01, *p* = 0.84; and mentalization: *β* = 0.36, *SE* = 0.07, *p* < 0.001).

### Predictors of Cognitive Disorganization

In the first regression model, we found that mindfulness was a significant predictor Cognitive Disorganization (*R*^2^ = 0.11, *β* = −0.3, *SE* = 0.05, *p* < 0.001). In the second step of the model, working memory and mindfulness were both included. However, the combination of these measures did not reveal significant differences as compared to the first regression model (*R*^2^ = 0.12; working memory: *β* = −0.03, *SE* = 0.06, *p* = 0.59; and mindfulness: *β* = −0.30, *SE* = 0.06, *p* < 0.001). In the final model, including mindfulness, working memory, and mentalization, only mentalization was a significant predictor of Cognitive Disorganization (*R*^2^ = 0.18; working memory: *β* = −0.05, *SE* = 0.06, *p* = 0.37; mindfulness: *β* = −0.11, *SE* = 0.07, *p* = 0.13; and mentalization: *β* = 0.36, *SE* = 0.07, *p* < 0.001).

### Predictors of Introverted Anhedonia

Similar to the case of Unusual Experiences and Cognitive Disorganization, mindfulness significantly predicted Introverted Anhedonia (*R*^2^ = 0.06, *β* = −0.25, *SE* = 0.07, *p* < 0.001). The addition of working memory to the model did not reveal a significant predictive effect of working memory (*R*^2^ = 0.07; working memory: *β* = −0.04, *SE* = 0.07, *p* = 0.59; mindfulness: *β* = −0.23, *SE* = 0.06, *p* < 0.001). In the final model, when mentalization was added to the regression model, the sole significant predictor was mentalization (*R*^2^ = 0.11; working memory: *β* = −0.06, *SE* = 0.06, *p* = 0.33; mindfulness: *β* = −0.07, *SE* = 0.07, *p* = 0.35; and mentalization: *β* = 0.27, *SE* = 0.07, *p* < 0.001).

### Predictors of Impulsive Nonconformity

In contrast to other schizotypy dimensions, Impulsive Nonconformity was not predicted by mindfulness and working memory (only mindfulness included in the model: *R*^2^ = 0.03, *β* = 0.05, *SE* = 0.06, *p* = 0.43; both working memory and mindfulness included in the model: *R*^2^ = 0.04; working memory: *β* = 0.06, *SE* = 0.06, *p* = 0.36; and mindfulness: *β* = −0.07, *SE* = 0.06, *p* = 0.27). However, the final model, including mindfulness, working memory, and mentalization, indicated a significant predictive effect of mentalization (*R*^2^ = 0.08; working memory *β* = 0.04, *SE* = 0.06, *p* = 0.50; mindfulness: *β* = 0.10, *SE* = 0.07, *p* = 0.17; and mentalization *β* = 0.28, *SE* = 0.07, *p* < 0.001).

## Discussion

Our main conclusion is that mentalization is a significant and common predictor of all schizotypal traits, including unusual experiences, cognitive disorganization, introverted anhedonia, and impulsive nonconformity. Furthermore, in line with our prior assumption, we found a significant correlation between mindfulness and mentalization, and low mindfulness and mentalization abilities were both associated with high levels of schizotypal features. However, in the regression analyses, mindfulness was not retained as a significant predictor of schizotypal traits when mentalization was added to the analysis because of the high covariance of mindfulness and mentalization.

Contrary to our hypothesis, working memory did not play a critical role in schizotypy: it yielded a weak correlation with cognitive disorganization and introverted anhedonia without significant predictive power. This is a counterintuitive finding because extensive research from schizophrenia and schizotypal personality disorder revealed impaired working memory (Moustafa et al., 2021). Meta-analytic evidence suggests a similarly impaired working memory in individuals at high clinical risk for psychosis (Zheng et al., 2018). However, the correlation between symptoms and working memory is less clear. The situation is even more dubious in non-clinical populations. Some studies demonstrated an association between letter-number sequencing and spatial working memory performances and schizotypy (Park et al., 1995; Matheson and Langdon, 2008). However, others did not find a link between schizotypy and verbal working memory (Lenzenweger and Gold, 2000). Remarkably, Louise et al. (2015) failed to detect a significant correlation between schizotypal traits and working memory: only a trend appeared between cognitive disorganization, impulsive nonconformity, and spatial span backward scores. The magnitude of correlation was like our results. However, more specific measures, such as inhibitory latency and cognitive flexibility on the Color-Word Interference Test, exhibited a more straightforward association with schizotypal traits, suggesting that future studies should use advanced testing strategies beyond conventional neuropsychological procedures (Louise et al., 2015).

In contrast to the weak association between schizotypal traits and working memory, we documented that mindfulness was associated with working memory. Indeed, several studies demonstrated that mindfulness training strengthens attention and working memory (Jha et al., 2019). Furthermore, working memory is crucially implicated in emotion regulation and decision making, so mindfulness training and improved working memory may be beneficial to cope with maladaptive schizotypal features, depression, and anxiety (Barkus, 2020).

The significant relationship between mentalization, mindfulness, and schizotypal personality traits is in line with the evidence from patients with various personality disorders (Fossati et al., 2012). Specifically, mindfulness predicted the overall number of personality disorder criteria in borderline and histrionic types. Deficient mindfulness might be the common denominator of several personality disorders (Fossati et al., 2012): it implies essential emotional awareness, a foundation of higher-level mentalization, and conscious reflection on mental states, which is a critical issue in borderline and schizotypal conditions (Marszal and Górska, 2015). Previous studies indicated that the regulative function of mindfulness and mentalization play a critical role in borderline personality organization (Marszal and Górska, 2015), and our results extend this model to schizotypy. Despite the symptom-level overlap, a notable difference between borderline personality and schizotypy may be the developmental disturbance of emotion regulation in individuals with borderline features, extending to interpersonal relationships, attachment, and identity coherence (Van Riel et al., 2017).

Since the pioneering hypothesis of Frith (1992), impaired mentalization has become an extensively documented phenomenon in psychosis-spectrum disorders, including individuals with a high clinical risk of schizophrenia, and in first-degree relatives of schizophrenia patients (Brüne, 2005; Debbané et al., 2016; Armando et al., 2019). However, much less is known about mindfulness. New evidence suggests that mindfulness has a predictive value in the physical health, psychological well-being, and end environmental quality of life of patients with schizophrenia-spectrum disorders (Bergmann et al., 2021). Although, as a supplementary treatment, mindfulness therapy is moderately effective in the clinical management of negative symptoms, depression, and social functions in schizophrenia (Khoury et al., 2013; Jansen et al., 2020), there is a surprising lack of studies exploring the characteristics of state- and trait-level mindfulness in schizophrenia patients. Nevertheless, mindfulness seems to be less helpful in reducing distress associated with frightening psychotic experiences (Strauss et al., 2015). It must be underlined that the relationship of mentalization, mindfulness, and working memory has not been investigated in clinically defined schizophrenia-spectrum disorders, and, therefore, our results cannot be generalized to clinical conditions.

As a stable foundation of mentalization, mindfulness opens a gate to mental states (Goodman, 2014). Consequently, mindfulness can enhance mentalization (Allen, 2013), a fundamental principle of Mentalizing Imagery Therapy. A case series recently showed that mental imagery and mindfulness focusing on attachment and interpersonal challenges could enhance mentalization and ameliorate depression and anxiety (Jain and Fonagy, 2020). These results are consistent with our cross-sectional data, demonstrating a positive relationship between mindfulness and mentalization. Moreover, according to Jain and Fonagy (2020), there is a causal link between mindfulness and mentalization: individuals can improve their mentalization skills by enhancing mindfulness.

Although our results suggest a straightforward relationship between mindfulness, mentalization, and schizotypy, we must consider several limitations. First, neither mindfulness nor mentalization is a unitary phenomenon. For example, the MZQ score can be subdivided into self-reflection, emotional awareness, psychic equivalence, and emotion regulation (Hausberg et al., 2012). However, the factor structure of MZQ is not consistent (Paridaens, 2017; Song and Choi, 2017), and our sample size was too small to increase the number of variables and separately investigate the predictive power of different mentalization types. Therefore, like other studies, we decided to include the total MZQ score as a single measure (Ballespi et al., 2018; Hayden et al., 2018; Probst et al., 2018). Future studies with larger samples may also investigate multiple facets of mindfulness (i.e., observing, describing, acting with awareness, non-judgmental, and non-reactive) and its relationship with schizotypy (Baer et al., 2006) by combining questionnaire data with results from structured interviews and real-life observations.

The relationship between working memory, mindfulness, mentalization, and schizotypy also requires additional studies using a more comprehensive array of tests. The WAIS-III working memory index taps on verbal and arithmetic functions (Lange, 2011), and it has not been elucidated how visual working memory is related to mindfulness, mentalization, and schizotypy. Based on the mechanism of Mentalizing Imagery Therapy (Jain and Fonagy, 2020), visual working memory may be more closely related to mindfulness and mentalization because visual working memory and mental imagery share the same cognitive and neuronal representations (Kosslyn et al., 2001).

In conclusion, we demonstrated that all schizotypy dimensions are associated with low levels of mindfulness and mentalization in the general population. As measured with the MAAS and MZQ, mindfulness and mentalization are highly interrelated, and mentalization is a better predictor of schizotypy than mindfulness. These results pose the possibility that therapeutic interventions focusing on both mentalization and mindfulness may be more effective than approaches targeting only one of them. Future studies are warranted to elucidate the relationship between schizotypy and different components of mindfulness and mentalization.

## Data Availability Statement

The raw data supporting the conclusions of this article will be made available by the authors, without undue reservation.

## Ethics Statement

Ethical review and approval was not required for the study on human participants in accordance with the local legislation and institutional requirements. Written informed consent for participation was not required for this study in accordance with the national legislation and the institutional requirements.

## Author Contributions

SK designed the study, coordinated data collection, and wrote the first draft of the paper. ET reviewed the relevant literature, performed the revision, and extension of the manuscript. ET and SK analyzed the data, reviewed, and edited the final version of the paper, which was approved by all authors. All authors contributed to the article and approved the submitted version.

## Funding

The study was supported by the BME-Biotechnology FIKP grant of EMMI (BME FIKP-BIO) and the National Research, Development, and Innovation Office (NKFI/OTKA K 128599).

## Conflict of Interest

The authors declare that the research was conducted in the absence of any commercial or financial relationships that could be construed as a potential conflict of interest.

## Publisher’s Note

All claims expressed in this article are solely those of the authors and do not necessarily represent those of their affiliated organizations, or those of the publisher, the editors and the reviewers. Any product that may be evaluated in this article, or claim that may be made by its manufacturer, is not guaranteed or endorsed by the publisher.
